# Reversal Effect of ALK Inhibitor NVP-TAE684 on ABCG2-Overexpressing Cancer Cells

**DOI:** 10.3389/fonc.2020.00228

**Published:** 2020-02-27

**Authors:** Jingqiu Wang, Jing-Quan Wang, Chao-Yun Cai, Qingbin Cui, Yuqi Yang, Zhuo-Xun Wu, Xingduo Dong, Leli Zeng, Linguo Zhao, Dong-Hua Yang, Zhe-Sheng Chen

**Affiliations:** ^1^Department of Pharmaceutical Sciences, College of Pharmacy and Health Sciences, St. John's University, Queens, NY, United States; ^2^College of Chemical Engineering, Nanjing Forestry University, Nanjing, China; ^3^School of Public Health, Guangzhou Medical University, Guangzhou, China; ^4^Tomas Lindahl Nobel Laureate Laboratory, Research Centre, The Seventh Affiliated Hospital, Sun Yat-sen University, Shenzhen, China

**Keywords:** NVP-TAE684, ATP-binding cassette (ABC) transporter, ABCG2, ALK inhibitor, multidrug resistance (MDR)

## Abstract

Failure of cancer chemotherapy is mostly due to multidrug resistance (MDR). Overcoming MDR mediated by overexpression of ATP binding cassette (ABC) transporters in cancer cells remains a big challenge. In this study, we explore whether NVP-TAE684, a novel ALK inhibitor which has the potential to inhibit the function of ABC transport, could reverse ABC transporter-mediated MDR. MTT assay was carried out to determine cell viability and reversal effect of NVP-TAE684 in parental and drug resistant cells. Drug accumulation and efflux assay was performed to examine the effect of NVP-TAE684 on the cellular accumulation and efflux of chemotherapeutic drugs. The ATPase activity of ABCG2 transporter in the presence or absence of NVP-TAE684 was conducted to determine the impact of NVP-TAE684 on ATP hydrolysis. Western blot analysis and immunofluorescence assay were used to investigate protein molecules related to MDR. In addition, the interaction between NVP-TAE684 and ABCG2 transporter was investigated via *in silico* analysis. MTT assay showed that NVP-TAE684 significantly decreased MDR caused byABCG2-, but not ABCC1-transporter. Drug accumulation and efflux tests indicated that the effect of NVP-TAE684 in decreasing MDR was due to the inhibition of efflux function of ABCG2 transporter. However, NVP-TAE684 did not alter the expression or change the subcellular localization of ABCG2 protein. Furthermore, ATPase activity analysis indicated that NVP-TAE684 could stimulate ABCG2 ATPase activity. Molecular *in silico* analysis showed that NVP-TAE684 interacts with the substrate binding sites of the ABCG2 transporter. Taken together, our study indicates that NVP-TAE684 could reduce the resistance of MDR cells to chemotherapeutic agents, which provides a promising strategy to overcome MDR.

## Introduction

Antineoplastic drugs can induce cancer cells resistant to treatment which makes the therapeutic effect of anti-cancer drugs greatly reduced and leads to multidrug resistance (MDR) (1). Classical MDR are mainly involved in drug-resistant proteins, which include the permeability-glycoprotein (P-gp/ABCB1) (2), multidrug resistance proteins (MRPs/ABCCs) (3), and breast cancer resistance protein (BCRP/MXR/ABCP/ABCG2) (4, 5).

P-gp, also known as MDR1 or ABCB1, is one of the most representative protein of ABC transporters (6–8). ABCB1 can identify various anti-cancer chemotherapeutic drugs, such as taxanes, camptothecins, anthracyclines, et al. (9). ATP hydrolysis provides energy for ABC transporters to extrude substrates out of tumor cells and lower the intracellular concentration of anticancer drugs, which results in weakening of the efficacy of chemotherapeutic drugs and eventually produces MDR (10).

The sub-family of MRPs/ABCCs is the C subgroup of ABC transporters. At present, nine MRPs with transport function have been found, ranging from MRP1 to MRP9 (11–13). Some of their structures (MRPs 4, 5, 7, 8, and 9) are similar to that of ABCB1. They contain two transmembrane regions and two ATP binding domains (14). But some of them have three transmembrane domains such as MRPs 1, 2, 3, and 6. MRP1/ABCC1 mediates the transport of anticancer drugs, including anthracyclines, methotrexate and doxorubicin (15–17).

ABCG2 is the first ABC semi-transporter found on cell membrane (18–20). Overexpression of ABCG2 can lead cancer cells resistant to various chemotherapeutic drugs. There are many overlaps between ABCG2, ABCB1, and ABCC1 in chemotherapeutic substrates, such as doxorubicin, epirubicin and mitoxantrone (21–23). However, the efflux capacity of ABCG2 for some chemotherapeutic drugs, such as vincristine and paclitaxel, is significantly lower than that of the other two drug-resistant proteins. In addition to chemotherapeutic drugs, ABCG2 has strong efflux ability to tyrosine kinase inhibitors, which can cause drug resistance in molecular targeted therapy. For example, imatinib and nilotinib for leukemia, sorafenib for liver cancer, erlotinib for NSCLC and lapatinib for HER2-positive breast cancer are substrates of ABCG2 (24–26). Therefore, screening the inhibitors of these three drug-resistant proteins is one of the effective methods to reverse MDR and improve efficacy of chemotherapy.

NVP-TAE684 is a selective ALK inhibitor which inhibits different downstream signaling transduction molecules in cancer cells, thereby down-regulating cell cycle and cell proliferation regulatory genes, resulting in arresting cell cycle, inhibiting cell proliferation and inducing cell apoptosis. NVP-TAE684 shows good anti-tumor effects to some mutant cells that are resistant to other ALK inhibitors (27–29). It was reported that NVP-TAE684 reverses MDR in human osteosarcoma by inhibiting ABCB1 function (30). However, whether NVP-TAE684 could affect other ABC transports has not been reported. In this study, we evaluate whether NVP-TAE684 can improve anticancer efficacy of drugs in ABCG2 or ABCC1 overexpressing MDR cells.

## Materials and Methods

### Chemicals

NVP-TAE684 was acquired from Chemie Tek (Indianapolis, IN). Fetal bovine serum (FBS), penicillin/streptomycin (P/S), Dulbecco's modified Eagle's Medium (DMEM), 0.25% trypsin and bovine serum albumin (BSA) were obtained from Corning Incorporated (Corning, NY). The GAPDH loading control monoclonal antibody (GA1R) (1 mg/mL, Cat # MA5-15738, lot #: SA247966), Alexa Fluor 488 conjugated goat anti-mouse IgG cross-adsorbed secondary antibody (2 mg/mL, Cat # A32723) were obtained from Thermo Fisher Scientific Inc (Rockford, IL). The anti-ABCG2 antibody, clone BXP-21 (Cat # MAB4146, lot #: 3026758) was obtained from Millipore (Billerica, MA). Horseradish peroxidase (HRP)-conjugated rabbit anti-mouse IgG secondary antibody (Cat # 7076S, lot #: 32) was obtained from Cell Signaling Technology Inc (Danvers, MA). Mitoxantrone, SN-38, topotecan, cisplatin, dimethylsulfoxide (DMSO), 3-(4,5-dimethylthiazolyl)-2,5-diphenyltetrazolium bromide (MTT), 4′,6-diamidino-2-phenylindole (DAPI), paraformaldehyde, Triton X-100 and other chemicals were purchased from Sigma Chemical Co (St. Louis, MO). Ko143 was purchased from Enzo Life Sciences (Farmingdale, NY). [^3^H]-Mitoxantrone (2.5 Ci/mmol) was purchased from Moravek Biochemicals, Inc (Brea, CA).

### Cell Lines and Cell Culture

The non-small cell lung cancer (NSCLC) NCI-H460 and its mitoxantrone-selected NCI-H460/MX20 cell line withABCG2 overexpression were used. NCI-H460/MX20 cell line was maintained in medium with 20 ng/mL mitoxantrone (31). The ABCG2-transfected HEK293 cell lines (HEK293/ABCG2-482-G2, HEK293/ABCG2-482-R2, and HEK293/ABCG2-482-T7) were transfected with full length ABCG2 coding arginine (R), glycine (G) or threonine (T) at 482 position, respectively. Its corresponding parental cell line, HEK293/pcDNA3.1, was transfected with an empty vector pcDNA3.1. All transfected cells were cultured with G418 at the concentration of 2 mg/mL. The human epidermal carcinoma cell line KB-3-1 and its ABCC1-overexpressing KB-CV60 cell line, were maintained in medium with 1 mg/mL of cepharanthine and 60 ng/mL of vincristine (32). Cells were grown in DMEM medium containing 10% FBS and 1% P/S, and kept in a 37°C humidified incubator supplied with 5% CO_2_. All drug-resistant cells were cultured in drug-free medium for more than 2 weeks before use.

### Cell Viability Examined by MTT Assay

MTT assay was used to measure the cell viability for the ABCG2 and ABCC1 reversal study as previously described (33). NCI-H460 and NCI-H460/MX20 cells, HEK293/pcDNA3.1 and HEK293/ABCG2 cells, KB-3-1 and KB-CV60 cells were used for the study. A total of 5 × 10^3^ cells were seeded into each well of a 96-well plate. On the next day, cells were treated with a serial concentrations of NVP-TAE684 for the toxicity test. For the reversal study, different concentrations of substrates were added 2 h after cells were pre-treated with NVP-TAE684, Ko143 (a positive reversal agent of ABCG2) or MK571(a positive reversal agent of ABCC1) at non-toxic concentrations. After 72 h treatment, MTT solution at 4 mg/mL was added and further incubated for 4 h. Finally, DMSO was added to each well after discarding the MTT solution. The OD values at 570 nm was determined with an accuSkan™ GO UV/Vis Microplate Spectrophotometer (Fisher Sci., Fair Lawn, NJ).

### Western Blotting

NCI-H460/MX20 cells were incubated with or without NVP-TAE684 (0.5 μM) for 0, 24, 48, and 72 h. Total protein was obtained by lysing cells on ice with lysis buffer (20 mM Tris-HCl pH 7.5, 150 mM NaCl, 1 mM Na_2_EDTA, 1 mM EGTA, 1% Triton, 2.5 mM sodium pyrophosphate, 1 mm β–glycerophosphate, 1 mM Na_3_VO_4_, and 1 μg/mL leupeptin). The protein concentrations of the cell lysates were determined using Pierce™ BCA Protein Assay Kit (Thermo Scientific, Rockford, IL) to ensure equal protein loading. SDS-polyacrylamide gel electrophoresis was used to separate the protein and then transferred onto the PVDF membrane. After 2 h blocking with non-fat milk, the membrane was incubated with primary antibody against ABCG2 or GAPDH (1:1000) at 4°C. On the next day, the membrane was incubated with HRP-labeled secondary antibody (1:1000) at room temperature for 2 h and detected by electrochemiluminescence. Photographs were taken and the relative band density was analyzed by Image J.

### Immunofluorescence Assay

NCI-H460 and NCI-H460/MX20 cells (2 × 10^4^ cells per well) were seeded into 24-well plates. Then NCI-H460/MX20 cells were incubated with 0.5 μM NVP-TAE684 for 0, 24, 48, and 72 h. After treatment with NVP-TAE684, cells were fixed in 4% polyformaldehyde, permeated with 0.25% Triton X-100. Then, 6% BSA was used to block the non-specific reaction. After incubation with antibody against ABCG2 (1:1000) overnight at 4°C. Cells were incubated with fluorescent IgG antibody (1:1000) in the dark for 2 h. DAPI (1 μg/mL) was used to stain nuclei of cells. A Nikon TE-2000S fluorescence microscopy (Nikon Instruments Inc., Melville, NY) was used to collect immunofluorescence images.

### ATPase Assay

As previously described (34), the ABCG2 membrane vesicles that overexpressed ABCG2 were from the protein extraction kit (Qproteome Plasma Membrane Protein Kit, Qiagen). Briefly, 20 μg ABCG2 membrane vesicles were incubated in assay buffer (containing pH 6.8 50 mM MES, 50 mM KCl, 5 mM sodium azide, 2 mM EGTA, 2 mM DTT, 1 mM ouabain, and 10 mM MgCl_2_). Then 0-40 μM NVP-TAE684 was incubated with these membrane vesicles for 3 min. The ATP hydrolysis was initialized by 5 mM of Mg-ATP, while 5% SDS solution was used to terminate the reaction. Subsequently, the light absorption at 880 nm was measured by the accuSkan GO UV/Vis Microplate Spectrophotometer.

### Accumulation and Efflux Assay

To determine the intracellular [^3^H]-mitoxantrone accumulation, 1 × 10^5^ NCI-H460 and NCI-H460/MX20 cells were inoculated into 24-well plates. On the next day, 0.2 and 0.5 μM NVP-TAE684 or 0.5 μM Ko143 were added 2 h before adding [^3^H]-mitoxantrone. After that, the cells were digested with trypsin to dissociate the cells into 5 mL scintillation solution. For [^3^H]-mitoxantrone efflux determination, 0.2 and 0.5 μM NVP-TAE684 or 0.5 μM Ko143 were added 2 h before adding [^3^H]-mitoxantrone. After that, the supernatant was discarded and medium was added with the absence or presence of inhibitor. Finally, cells were collected at 0, 30, 60, and 120 min. Packard TRICARB 1900CA liquid scintillation analyzer (Downers Grove, IL) was used to measure the radioactivity.

### Molecular Docking Analysis

The molecular docking analysis was conducted in Maestro v11.1 (Schrödinger, LLC) by the default protocols (35). The ligand NVP-TAE684 was prepared, then ABCG2 protein (Protein Data Bank ID: 6FFC) (36) was prepared. The ABCG2 protein obtained was bound to a synthetic derivative of ABCG2 inhibitor Ko143 (36). The docking grid was generated based on the position of the Ko143 derivative with the default protocol. Subsequently, glide docking was performed and induce-fit docking was conducted based on the results of glide docking.

### Statistical Analysis

All data were presented as the mean ± SD. All experiments were done independently at least three times. One-way ANOVA was used to analyze the difference between control and experimental group. It was considered as significant when *p*-value is < 0.05.

## Results

### Reversal Effect of NVP-TAE684 on the ABCG2-Mediated Drug-Resistant Cells

**Figure 1:**
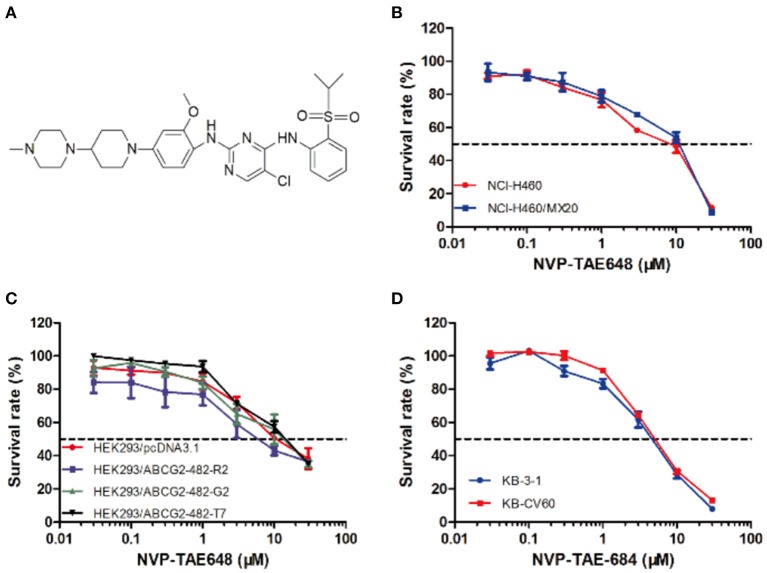
Structure of NVP-TAE684 and its cytotoxicity in sensitive, ABCG2-, and ABCC1-mediated MDR cells. **(A)** Chemical structure of NVP-TAE684. **(B)** Survival rate of mitoxantrone-selected NCI-H460/MX20 and its parental cells treated with NVP-TAE684 for 72 h. **(C)** Survival rate of empty vector or full length ABCG2-transfected HEK293 cells after 72 h treatment with NVP-TAE684. **(D)** Survival rate of KB-3-1 and KB-CV60 cells treated with NVP-TAE684 for 72 h.

We examined the cytotoxicity of NVP-TAE684 on cells overexpressed ABCG2 or ABCC1. As shown in Figure 1, NVP-TAE684 showed non-toxicity at low concentrations. Therefore, based on this result, we selected non-toxic concentrations of NVP-TAE684, which are 0.2 μM and 0.5 μM, for the following studies.

As shown in Table 1, the IC_50_ values of several known ABCG2 substrates (mitoxantrone, SN-38 and topotecan) in NCI-H460/MX20 cells was concentration-dependently decreased by NVP-TAE684 compared with their control cells. Also, the efficacy of above substrates in ABCG2-gene-transfected cells compared with that in empty vector transfectant cells was significantly increased after co-cultured with NVP-TAE684 (Table 2). However, the cytotoxicity of cisplatin, which is not a substrate of ABCG2, was not significantly affected by NVP-TAE684. These results indicated that NVP-TAE684 could antagonize MDR mediated by ABCG2-overexpression. As shown in Table 3, the IC_50_ value of vincristine in KB-CV60 cells was not significantly reduced by NVP-TAE684, which indicated that NVP-TAE684 could not reverse MDR mediated by ABCC1.

**Table 1:** NVP-TAE684 lowered the IC_50_ values of anticancer agents in NCI-H460/MX20 cells.

**Treatment**	**IC_50_ ± SD^a^ (RF^b^) (μM)**	
	**NCI-H460**	**NCI-H460/MX20**
Mitoxantrone	0.026 ± 0.009 (1.00)	2.783 ± 0.047 (105.40)
+NVP-TAE684 (0.2 μM)	0.023 ± 0.018 (0.88)	0.227 ± 0.057 (8.61)^*^
+NVP-TAE684 (0.5 μM)	0.019 ± 0.002 (0.73)	0.115 ± 0.056 (4.34)^*^
+Ko 143 (0.5 μM)	0.026 ±0.012 (0.99)	0.123 ±0.041 (4.67)^*^
SN-38	0.039 ± 0.020 (1.00)	2.547 ± 0.138 (67.29)
+NVP-TAE684 (0.2 μM)	0.025 ± 0.021 (0.65)	0.113 ± 0.091 (2.98)^*^
+NVP-TAE684 (0.5 μM)	0.022 ± 0.013 (0.59)	0.089 ± 0.010 (2.35)^*^
+Ko 143 (0.5 μM)	0.032 ± 0.022 (0.85)	0.090 ± 0.010 (2.38)^*^
Topotecan	0.042 ± 0.006 (1.00)	3.486 ± 0.110 (83.66)
+NVP-TAE684 (0.2 μM)	0.037 ± 0.009 (0.88)	0.186 ± 0.099 (4.45)^*^
+NVP-TAE684 (0.5 μM)	0.030 ± 0.009 (0.71)	0.108 ±0.079 (2.60)^*^
+Ko 143 (0.5 μM)	0.062 ± 0.010 (1.49)	0.119 ± 0.068 (3.85)^*^
Cisplatin	2.155 ± 0.075 (1.00)	2.704 ± 0.068 (1.25)
+NVP-TAE684 (0.2 μM)	2.573 ± 0.063 (1.19)	2.703 ± 0.046 (1.25)
+NVP-TAE684 (0.5 μM)	2.673 ± 0.067 (1.24)	2.907 ± 0.088 (1.35)
+Ko 143 (0.5 μM)	2.931 ± 0.066 (1.36)	2.636 ± 0.090 (1.22)

a *IC_50_ values represent the mean ± SD obtained from three independent experiments*.

b *Resistance fold (RF) was calculated by dividing the IC_50_ values of NCI-H460/MX20 cell by the IC_50_ of NCI-H460 cell in the presence or absence of NVP-TAE684 or positive control inhibitor*.

* *Indicates p < 0.05 vs. cells treated with antineoplastic drug only*.

**Table 2:** NVP-TAE684 lowered the IC_50_ values of anticancer drugs in ABCG2-gene-transfected cells.

**Treatment**	**IC_50_ ± SD^a^ (RF^b^) (μM)**			
	**HEK293/pcDNA3.1**	**HEK293/ABCG2-482-G2**	**HEK293/ABCG2-482-R2**	**HEK293/ABCG2-482-T7**
Mitoxantrone	0.053 ± 0.014 (1.00)	0.928 ± 0.199 (17.36)	1.077 ± 0.340 (20.15)	1.197 ± 0.098 (22.40)
+NVP-TAE684 (0.2 μM)	0.080 ± 0.011 (1.49)	0.440 ± 0.082 (8.23)^*^	0.416 ± 0.164 (7.79)^*^	0.548 ± 0.042 (10.25)^*^
+NVP-TAE684 (0.5 μM)	0.060 ± 0.007 (1.12)	0.099 ± 0.018 (1.87)^*^	0.221 ± 0.016 (4.13)^*^	0.329 ± 0.118 (6.15)^*^
+Ko 143 (0.5 μM)	0.072 ± 0.010 (1.35)	0.110 ± 0.069 (2.20)^*^	0.171 ± 0.024 (3.20)^*^	0.351 ± 0.052 (6.57)^*^
SN-38	0.072 ± 0.012 (1.00)	1.577 ± 0.152 (21.82)	1.590 ± 0.119 (22.00)	1.600 ± 0.131 (22.14)
+NVP-TAE684 (0.2 μM)	0.085 ± 0.013 (1.18)	0.604 ± 0.163 (8.35)^*^	0.477 ± 0.115 (6.60)^*^	0.583 ± 0.153 (8.07)^*^
+NVP-TAE684 (0.5 μM)	0.068 ± 0.012 (0.94)	0.117 ± 0.011 (1.16)^*^	0.138 ± 0.054 (1.91)^*^	0.267 ± 0.152 (3.70)^*^
+Ko 143 (0.5 μM)	0.089 ± 0.011 (1.24)	0.387 ± 0.018 (5.36)^*^	0.397 ± 0.083 (5.49)^*^	0.469 ± 0.164 (6.49)^*^
Topotecan	0.032 ± 0.01 (1.00)	0.858 ± 0.142 (26.40)	0.659 ± 0.116 (20.28)	0.966 ± 0.158 (29.73)
+NVP-TAE684 (0.2 μM)	0.026 ± 0.012 (0.84)	0.232 ± 0.187 (7.16)^*^	0.110 ± 0.016 (3.40)^*^	0.281 ± 0.024 (8.64)^*^
+NVP-TAE684 (0.5 μM)	0.023 ± 0.008 (0.71)	0.058 ± 0.031 (1.79)^*^	0.055 ± 0.011 (1.68)^*^	0.142 ± 0.044 (4.37)^*^
+Ko 143 (0.5 μM)	0.022 ± 0.012 (0.69)	0.114 ± 0.089 (3.52)^*^	0.059 ± 0.012 (1.81)^*^	0.170 ± 0.160 (5.23)^*^
Cisplatin	1.616 ± 0.750 (1.00)	1.768 ± 0.751 (1.09)	2.086 ± 1.147 (1.29)	1.547 ± 0.876 (0.96)
+NVP-TAE684 (0.2 μM)	1.915 ± 0.487 (1.18)	1.712 ± 0.897 (1.06)	2.312 ± 1.426 (1.43)	1.557 ± 0.984 (0.93)
+NVP-TAE684 (0.5 μM)	2.009 ± 0.669 (1.24)	2.107 ± 0.715 (1.30)	1.987 ± 1.359 (1.23)	1.408 ± 0.781 (0.87)
+Ko 143 (0.5 μM)	2.191 ± 0.55 (1.35)	1.926 ± 0.542 (1.19)	1.868 ± 1.186 (1.16)	1.668 ± 1.072 (1.03)

a *IC_50_ values represent the mean ± SD obtained from three independent experiments*.

b *Resistance fold (RF) was calculated from dividing the IC_50_ values of HEK293/ABCG2 cells by the IC_50_ of HEK293/pcDNA3.1 cells in the presence or absence of NVP-TAE684 or positive control inhibitor*.

* *Indicates p < 0.05 vs. cells treated with antineoplastic drug only*.

**Table 3:** NVP-TAE684 does not reverse MDR mediated by ABCC1.

**Treatment**	IC_50_ ± SD^a^ (RF^b^) (μM)	
	**KB-3-1**	**KB-CV60**
Vincristine	0.075 ± 0.019 (1.00)	1.383 ± 0.060 (18.44)
+NVP-TAE684 (0.2 μM)	0.085 ± 0.013 (1.14)	1.447 ± 0.066 (19.30)
+NVP-TAE684 (0.5 μM)	0.063 ± 0.017 (0.82)	1.168 ± 0.078 (15.58)
+MK571 (25 μM)	0.067 ± 0.015 (0.90)	0.347 ± 0.121 (4.63)^*^
Cisplatin	1.398 ± 0.120 (1.00)	1.457 ± 0.093 (1.04)
+NVP-TAE684 (0.2 μM)	1.475 ± 0.150 (1.06)	1.568 ± 0.071 (1.12)
+NVP-TAE684 (0.5 μM)	1.175 ± 0.081 (0.84)	1.287 ± 0.141 (0.92)
+MK571 (25 μM)	1.327 ± 0.084 (0.95)	1.437 ± 0.072 (1.03)

a *IC_50_ values represent the mean ± SD obtained from three independent experiments*.

b *Resistance fold (RF) was calculated from dividing the IC_50_ values of KB-CV60 cells by the IC_50_ of KB-3-1 cell line in the presence or absence of NVP-TAE684 or positive control inhibitor*.

* *Indicates p < 0.05 vs. group treated with antineoplastic drug only*.

### NVP-TAE684 Does Not Change the Protein Expression or Localization of ABCG2

**Figure 2:**
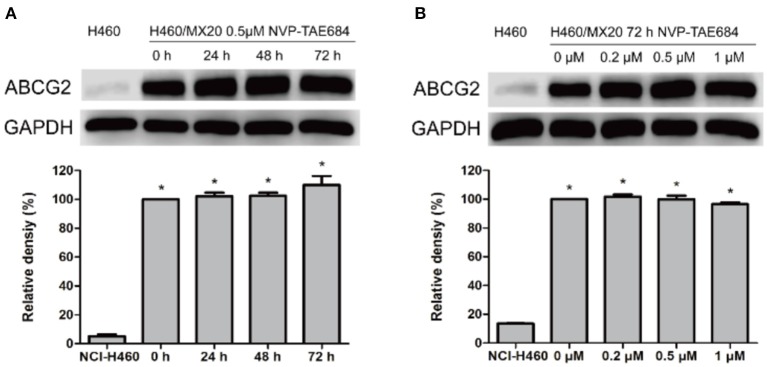
The protein expression of ABCG2 was not changed after co-incubation with NVP-TAE684. **(A)** ABCG2 expression after treatment with 0.5 μM NVP-TAE684 for 0, 24, 48, and 72 h. **(B)** ABCG2 protein expression after treatment with 0.2, 0.5, 1 μM NVP-TAE684 for 72 h. Image J was used to quantify the relative band density. *p < 0.05, compared with control group.

**Figure 3:**
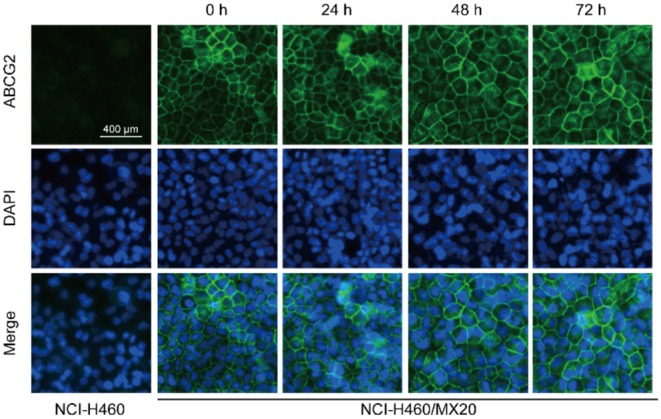
Subcellular localization of ABCG2 was not changed after treatment with NVP-TAE684 at 0.5 μM.

Western blotting and immunofluorescence analysis were performed to examine the expression and subcellular localization of ABCG2. According to the results in Figure 2, after incubation with 0.5 μM NVP-TAE684 for 24, 48, and 72 h, the expression of ABCG2 (72 kDa) was not altered. In addition, the ABCG2 expression remained unchanged after incubation with 0.2, 0.5, and 1 μM of NVP-TAE684 for up to 72 h. Furthermore, as shown in Figure 3, the localization of ABCG2 transporter was remained on cell membrane after treated with NVP-TAE684 at indicated concentration for 0, 24, 48, 72 h.

### NVP-TAE684 Increased the [^3^H]-Mitoxantrone Intracellular Accumulation in NCI-H460/MX20 Cells

**Figure 4:**
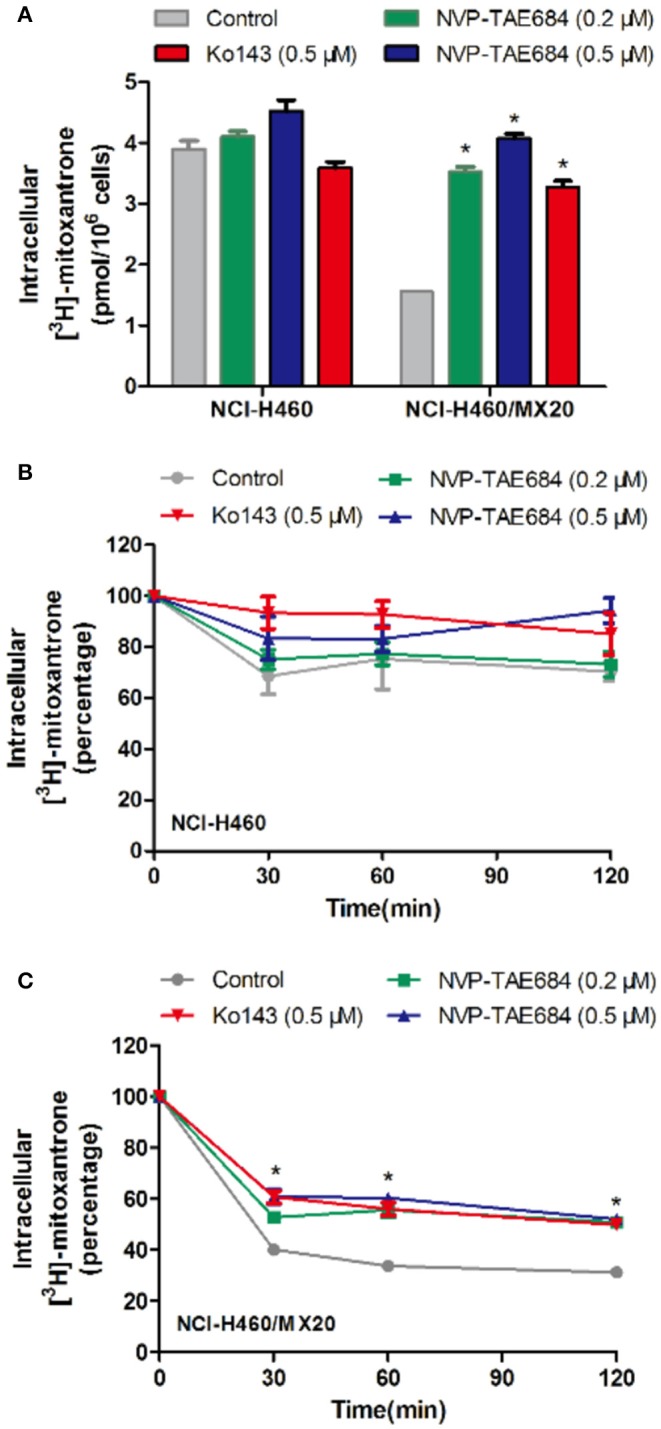
NVP-TAE684 inhibited the efflux function of ABCG2 which resulted in increasing intracellular concentration of [^3^H]-mitoxantrone. **(A)** The effect of NVP-TAE684 on the [^3^H]-mitoxantrone accumulation in MDR cells. **(B,C)** The effects of NVP-TAE684 on the efflux activity mediated by ABCG2 in NCI- H460/MX20 and NCI-H460 cells. Ko143 served as a reference inhibitor of ABCG2. *p < 0.05, compared with control group.

To understand the mechanism of action of NVP-TAE684 for reversal activity, drug accumulation assay was conducted to evaluate the effect of NVP-TAE684 on the [^3^H]-mitoxantrone accumulation in sensitive and drug-resistant cells. It was found that NVP-TAE684 had the ability to significantly increase the intracellular concentration of [^3^H]-mitoxantrone in ABCG2 overexpression cells, while NVP-TAE684 did not have impact on the [^3^H]-mitoxantrone accumulation in its parental NCI-H460 cells (Figure 4A).

### The Efflux Activity of ABCG2 Was Inhibited by NVP-TAE684 in NCI-H460/MX20 Cells

Since ABCG2 transporter can pump out drugs, drug efflux assay was used to evaluate whether NVP-TAE684 can affect the efflux function of ABCG2 transporter. It was found that NVP-TAE684 significantly reduced the extrusion of [^3^H]-mitoxantrone in NCI-H460/MX20 cells, but it had no significant effect on the efflux function mediated by ABCG2 in corresponding parental cells. These data demonstrated that NVP-TAE684 can impede the efflux activity of ABCG2 transporter which resulted in increasing the intracellular accumulation of anticancer drugs (Figures 4B,C).

### NVP-TAE684 Stimulated the ABCG2 ATPase Activity

**Figure 5:**
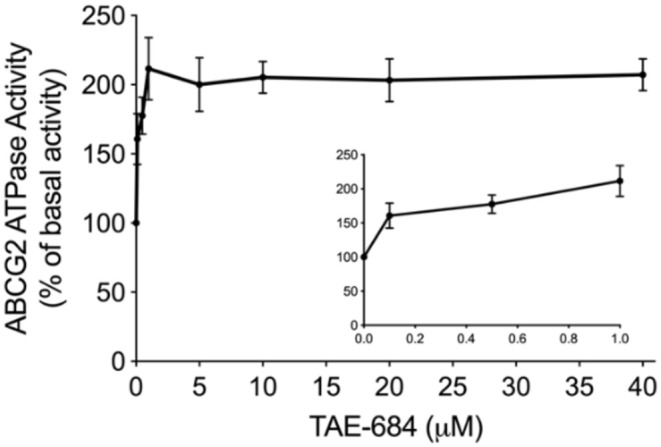
NVP-TAE684 stimulates the activity of ABCG2 ATPase. Data are mean ± SD, representatives of three independent experiments.

To determine the effect of NVP-TAE684 on ABCG2 ATPase activity, an ATPase assay kit was used to measure the ABCG2-mediated ATP hydrolysis in membrane vesicles after incubation with a serial concentrations of NVP-TAE684. According to Figure 5, the ATPase activity of ABCG2 transporter was stimulated by NVP-TAE684 in a concentration-dependent pattern. ATPase activity reached a maximum of 211.6% of the basal activity for ABCG2. The stimulatory effect of NVP-TAE684 reached 50% maximal (EC_50_) at 0.091 μM for ABCG2.

### Molecular Docking Analysis on the Interaction of NVP-TAE684 and ABCG2

**Figure 6:**
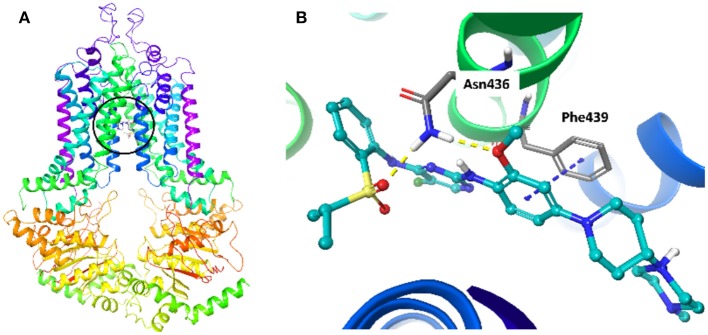
The induced fit docking analysis of NVP-TAE684 and ABCG2 protein (PDB: 6FFC). **(A)** The binding site of NVP-TAE684 with ABCG2 protein was indicated with a circle. The ABCG2 protein was shown as ribbons. **(B)** The predicted binding mode of NVP-TAE684 and ABCG2 protein. Hydrogen bonds and π-π stacking were indicated with yellow and blue dot line, respectively. The atoms of NVP-TAE684 was colored as follows: carbon-cyan, hydrogen-white, oxygen-red, nitrogen-blue, fluoride-green, sulfur-yellow.

To explore the interaction between NVP-TAE684 and ABCG2, a molecular docking analysis was performed. The docked position of NVP-TAE684 and ABCG2 protein with highest docking score (-12.929 kcal/mol) was shown in Figure 6. Both hydrogen bonds and π-π interaction are included in the interaction of NVP-TAE684 and ABCG2 protein: π-π interaction between the methoxy phenyl group of NVP-TAE684 and the residue Phe439; hydrogen bonds formed between the residue Asn436 and the sulfonylphenyl or methoxy groups of NVP-TAE684.

## Discussion

ABCG2 protein is a member of ABC transporters (37). ABCG2 overexpression can lead to MDR. Substrates of ABCG2 include anthracyclines, camptothecins and methotrexate (38). Since ABCG2 is an important contributor to MDR, inhibiting ABCG2 activity may help improve the efficacy of chemotherapeutic drugs. At present, various specific and non-specific inhibitors of ABCG2 have been found. FTC is a mycotoxin isolated from Aspergillus fumigatus (39, 40). It can specifically sensitize chemotherapeutic agents to MDR mediated by ABCG2. However, its use *in vivo* is limited due to its neurotoxic effect. FTC tetracyclic analog Ko143 could interrupt the efflux activity of ABCG2 transporter (41), which has no toxicity in mice with high oral dose. Ko143 is the first specific ABCG2 inhibitor suitable for use *in vivo* (42). However, Ko143 is rapidly metabolized into a compound that is ineffective for clinical use (35). Thus, it is important to find more effective and non-toxic ABCG2 reversal agents. Recent research reported that some ALK inhibitors could sensitize chemotherapeutic drugs to ABC-mediated MDR (7, 43, 44). NVP-TAE684 is an ALK inhibitor which was reported that it could inhibit ABCB1 transporter function, but whether NVP-TAE684 could inhibit ABCG2 or ABCC1 transporter has not been reported. In this study, we explore whether NVP-TAE684 could reverse ABCG2- or ABCC1-mediated MDR and the results showed that NVP-TAE684 had reversal effects on ABCG2-mediated drug-resistant cells but showed no reversal effect on ABCC1-mediated MDR.

First, we tested the cytotoxicity of NVP-TAE684 and we found that NVP-TAE684 showed low toxicity at low concentrations. We selected two non-toxic concentrations for reversal study. MTT results showed that IC_50_ values of several known ABCG2 substrates, including mitoxantrone, SN-38 and topotecan, in mitoxantrone-selected NCI-H460/MX20 cells reduced upon the treatment by NVP-TAE684 at non-toxicity concentrations. To confirm that the reversal effect was related to ABCG2, we determined the effect of NVP-TAE684 on ABCG2-gene-transfected HEK293 cells. We found that NVP-TAE684 showed reversal effect on HEK293/ABCG2 cells. However, the cytotoxicity of cisplatin, which is a non-substrate of ABCG2 (45), was not altered by NVP-TAE684. Moreover, we evaluated the reversal effect of NVP-TAE684 on ABCC1-overexpressing KB-CV60 cells and found that NVP-TAE684 had no significant reversal effect on KB-CV60 cells. These results indicated that NVP-TAE684 was specific to the substrates of the ABCG2 transporter.

Downregulating ABCG2 expression may lead to reversing MDR. In order to understand whether NVP-TAE684 affects either protein expression or localization of ABCG2, immunofluorescence and Western blotting experiments were carried out. Immunofluorescence results showed that the subcellular localization of ABCG2 transporter was unchanged when the cells were cultured with NVP-TAE684. In addition, immunoblotting results indicated that NVP-TAE684 did not downregulate the expression of ABCG2 transporter after up to 72 h treatment. Therefore, NVP-TAE684 did not change the expression of ABCG2 transporter and its subcellular localization.

It has been found that cancer cells can pump antineoplastic drugs out of cells through a complicated efflux pump system, thereby reducing intracellular concentration of many structurally unrelated anticancer drugs and leading to MDR (46, 47). According to our results, the intracellular concentration of tritium-labeled antineoplastic drug in MDR cells was significantly increased by NVP-TAE684 treatment, while no change was found in corresponding parental cells. Further assay suggested that NVP-TAE684 significantly reduced the efflux of tritium-labeled chemotherapeutic agent in drug resistant cells. These results showed that NVP-TAE684 has inhibitory activity on efflux activity of ABCG2 transporter and it resulted in increasing the accumulation of antineoplastic agents. Since ABCG2 has an ATP-binding region that is essential for substrate transport, and the function of ABCG2 transporter relies on the energy from the hydrolysis of ATP by the transporter, which can be modulated by the presence of substrates or inhibitors. Thus, monitoring ATPase activity allows for identification of those compounds that interact with ABCG2. ABCG2 exhibits a drug-dependent ATP hydrolysis activity, and a variety of ABCG2 inhibitors, as well as ABCG2 substrates, can either stimulate or inhibit ATPase activity (48). The results showed that the activity of ABCG2 ATPase was stimulated by NVP-TAE684, suggesting that NVP-TAE684 could act as a substrate, which may competitively occupy the drug binding site of ABCG2 transporter. Molecular docking study showed that NVP-TAE684 can interact with the transmembrane domain of ABCG2 with a docking score of−12.929 kcal/mol, and the interaction between NVP-TAE684 and ABCG2 proteins includes π-π interaction and hydrogen bond. This indicates that NVP-TAE684 has a strong direct interaction with ABCG2. Therefore, the reversal effect of ALK inhibitor NVP-TAE684 was related to its inhibition on drug efflux function probably by competitively occupy the substrate binding site of ABCG2 transporter.

## Conclusion

This study suggests that NVP-TAE684 reverses ABCG2-mediated MDR by inhibiting the efflux activity of cancer cells, therefore increasing intracellular concentration of chemotherapeutic drugs. Our study provides a rationale for the combinational use of NVP-TAE684 and ABCG2-substrate drugs to circumvent ABCG2-mediated MDR.

## Data Availability Statement

All datasets generated for this study are included in the article/supplementary material.

## Author Contributions

Conceptualization: D-HY and Z-SC. Methodology: JW, C-YC, QC, J-QW, YY, Z-XW, XD, and LZe. Writing—original draft preparation: JW. Writing, review, and editing: JW, D-HY, Z-SC, and LZh. Supervision: Z-SC and D-HY. Funding acquisition: Z-SC and LZh.

### Conflict of Interest

The authors declare that the research was conducted in the absence of any commercial or financial relationships that could be construed as a potential conflict of interest.
